# Supplementary material for: qDSB-Seq is a general method for genome-wide quantification of DNA double-strand breaks using sequencing
Source: Nat Commun. 2019 May 24;10:2313. doi: 10.1038/s41467-019-10332-8 (PMC6534554; doi:10.1038/s41467-019-10332-8)
Supplement: Supplementary file 1 — Supplementary Information [file 41467_2019_10332_MOESM1_ESM.pdf]

Supplementary Information

qDSB-Seq is a general method for genome-wide quantification of DNA double-strand breaks using sequencing

Zhu, Biernacka *et al*.

Table of Contents

Supplementary Figures 1 to 6

Supplementary Tables 1 to 4

Supplementary Figures

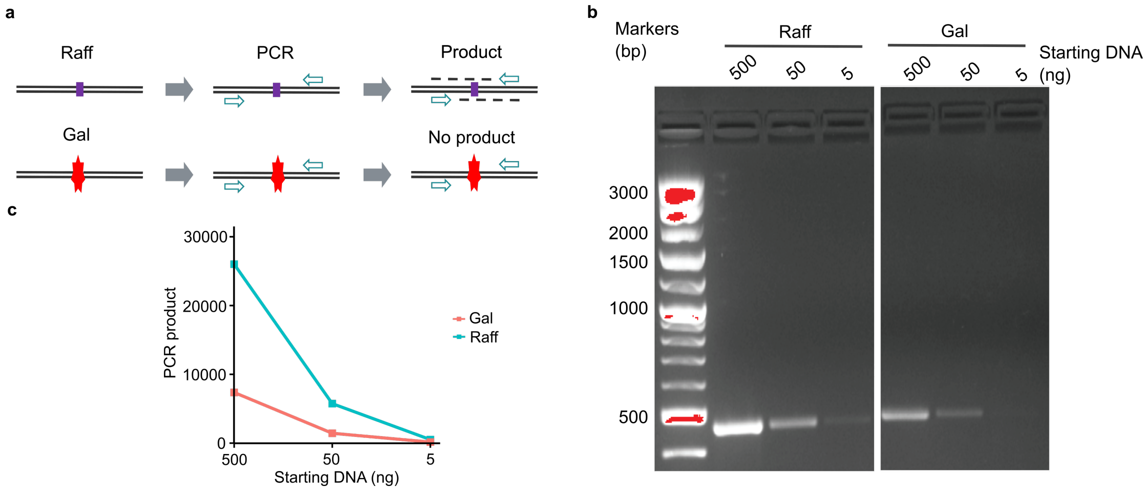

Supplementary Figure 1. Estimation of cutting efficiency using semi-quantitative PCR (sqPCR). **(a)** A schema for sqPCR design and its results for cells cultured in raffinose (no I-SceI digestion) and galactose (I-SceI digestion). The purple rectangle and red star represent I-SceI recognition site and the digestion of I-SceI recognition sequence induced by galactose addition, respectively. **(b)** Gel electrophoresis of PCR products in Raff and Gal samples for different amount of starting DNA. **(c)** Quantification of PCR products based on electrophoresis results. Source data are provided as a Source Data file.

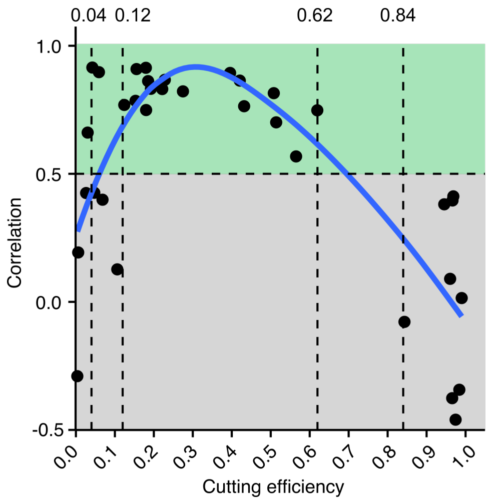

Supplementary Figure 2. Pearson correlation between cutting efficiencies and the labeled reads for all sites recognized by a given enzyme was calculated for samples treated with restriction enzymes with multiple cutting sites (35 separate digestions). The green and grey areas were divided by correlation 0.5 that shows strong relationship between cutting efficiencies and the labeled reads for quantification. Source data are provided as a Source Data file.

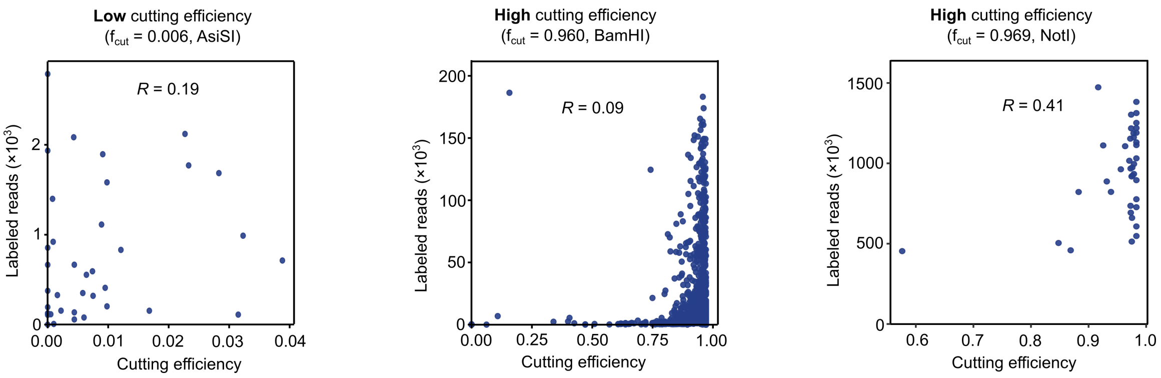

Supplementary Figure 3. Employment of extremely low or high cutting efficiencies results in loss of proportional relationship between the labeled reads and cutting efficiencies at enzyme cutting sites. Pearson correlation (*R*) between cutting efficiencies and the labeled reads was calculated for all sites recognized by a given enzyme. From left to right, AsiSI, BamHI, and NotI-treated samples are shown.

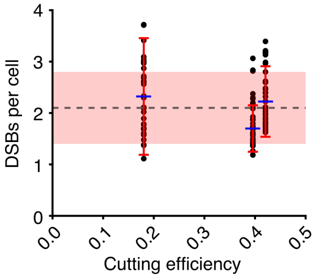

Supplementary Figure 4. Quantification of DSBs in G_1 *pif1-m2* mutant cells. The dashed gray line and the pink area are, respectively, the mean value (n = 3) and 95% confidence interval for all three biological replicates. DSBs per cell values for individual samples are denoted by blue dash and described in Methods, SD values are denoted by red intervals (n = 39 for each sample). Source data are provided as a Source Data file.

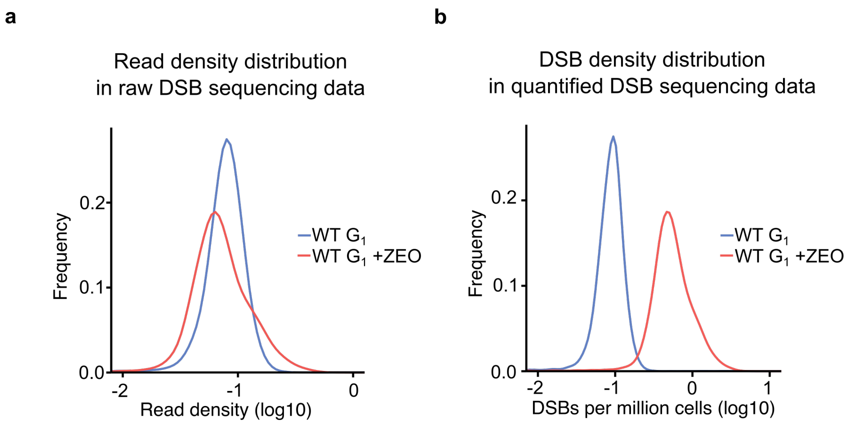

Supplementary Figure 5. Density plot of raw and quantified DSB sequencing data for Zeocin-treated and untreated G_1 samples. **(a)** Read density was defined as the number of i-BLESS reads mapped to a given region, divided by region length, here a 500 bp sliding window and a 50 bp step. **(b)** DSB density distribution. DSB density was calculated from quantified DSB numbers per cell using a 500 bp sliding window, divided by region length.

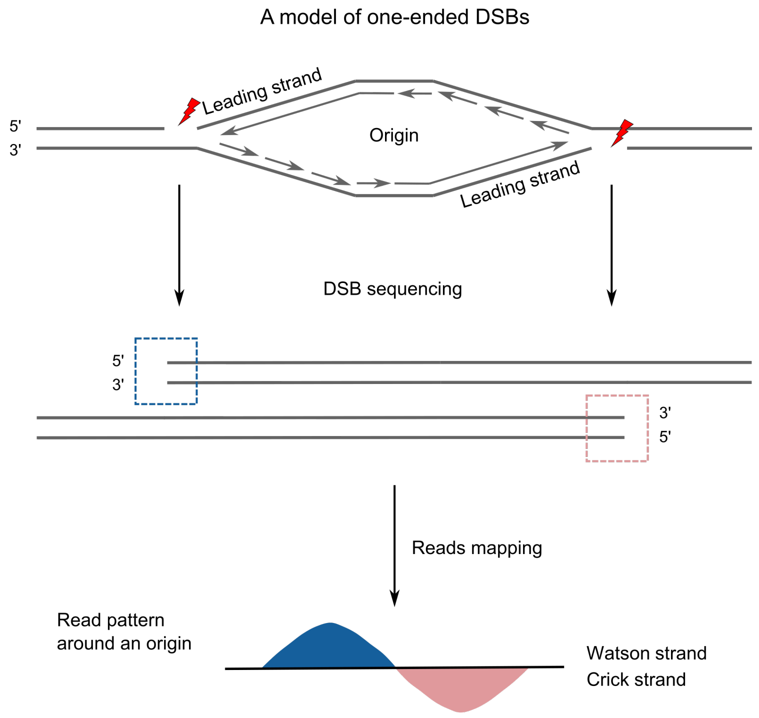

Supplementary Figure 6. A characteristic pattern of i-BLESS reads observed for one-ended DSBs. The pattern arises around replication origins due to broken replication forks resulting in one-ended DSBs.

Supplementary Tables

Supplementary Table 1. Pearson correlation between i-BLESS labeled reads and cutting efficiencies from a restriction enzyme.

**Enzyme**	**Sample name**	**Average cutting efficiency**	***R***
NotI	G_1 exp6	0.04	0.915
NotI	G_1 exp1	0.18	0.914
NotI	ZEO	0.16	0.909
SrfI	G_1 exp6	0.06	0.897
NotI	G_1 pif1 exp2	0.40	0.894
NotI	S exp4	0.23	0.867
NotI	G_1 pif1 exp3	0.42	0.864
NotI	S exp1	0.18	0.862
NotI	S exp2	0.19	0.832
NotI	G_1 exp7	0.22	0.831
NotI	S exp6	0.28	0.822
NotI	G_1 exp10	0.51	0.815
NotI	WT S	0.15	0.786
NotI	WT +CPT	0.12	0.769
NotI	G_1 exp8	0.43	0.764
NotI	G_1 pif1 exp1	0.18	0.749
NotI	WT +HU	0.62	0.748
NotI	G_1 exp9	0.51	0.701
AsiSI	G_1 exp4	0.03	0.661
NotI	G_1 exp7	0.56	0.568
NotI	S exp5	0.05	0.426
NotI	S exp3	0.03	0.425
NotI	G_1 exp2	0.97	0.412
SrfI	G_1 exp2	0.07	0.399
NotI	G_1 exp3	0.97	0.396
NotI	G_1 exp5	0.94	0.381
AsiSI	G_1 exp2	0.006	0.193
SrfI	G_1 exp3	0.11	0.127
BamHI	BamHI	0.96	0.090

NotI	G_1 exp4	0.99	0.015
SrfI	G_1 exp4	0.84	-0.078
AsiSI	G_1 exp3	0.004	-0.290
SrfI	G_1 exp5	0.98	-0.343
AsiSI	G_1 exp5	0.97	-0.376
AsiSI	G_1 exp6	0.98	-0.460

Note: only filtered cutting sites were used for calculating Pearson correlation as described in Methods. The results are sorted according to descending *R*.

Supplementary Table 2. Absolute DSB frequencies per cell and their variation influenced by low and high cutting efficiencies in G_1 untreated samples. Source data are provided as a Source Data file.

Sample name	Enzyme	Average cutting efficiency	Studied DSBs	SD from multiple cutting sites	SD / Studied DSBs	Number of enzyme cutting sites per Mbases	% of enzyme-induced spike-in reads
**Adequate cutting efficiency**							
G_1 exp6	NotI	0.04	0.7	0.6	0.84	3	20
G_1 exp6	SrfI	0.06	0.8	0.4	0.49	2	11
G_1 exp2	SrfI	0.07	0.6	0.2	0.41	2	20
G_1 exp3	SrfI	0.11	1.7	0.7	0.44	2	20
G_1 exp1	NotI	0.18	1.1	0.3	0.25	3	87
Mean			1.0				
SD			0.4				
G_1 pif1 exp1	NotI	0.18	2.3	1.1	0.49	3	75
G_1 pif1 exp2	NotI	0.40	1.7	0.5	0.27	3	90
G_1 pif1 exp3	NotI	0.42	2.2	0.7	0.31	3	88
Mean			2.1				
SD			0.3				
**Low cutting efficiency**							
G_1 exp3	AsiSI	0.004	6774	1770	0.26	3	0
G_1 exp2	AsiSI	0.006	39	375	9.65	3	0
G_1 exp4	AsiSI	0.03	9.5	16.0	1.69	3	1
Mean			2274				
SD			3897				
**High cutting efficiency**							
G_1 exp4	SrfI	0.84	1.3	0.5	0.38	2	57
G_1 exp5	NotI	0.95	4.0	1.9	0.47	3	22
G_1 exp15	BamHI	0.96	2.2	27	12.40	139	93
G_1 exp5	AsiSI	0.97	7.9	17	2.20	3	22
G_1 exp3	NotI	0.97	11	4.1	0.38	3	62
G_1 exp2	NotI	0.97	5.6	1.9	0.33	3	69
G_1 exp6	AsiSI	0.98	5.2	2.5	0.48	3	57
G_1 exp5	SrfI	0.98	4.0	0.9	0.22	2	13

G_1 exp4	NotI	0.99	6.9	3.4	0.49	3	31
Mean			5.3				
SD			2.9				

Supplementary Table 3. DSB frequencies per cell near Replication Fork Barriers (RFBs) in an rDNA array.

	rDSB-1	rDSB-2	rDSB-3	DSBs on RFBs	SD of DSBs on RFBs
WT G_1	0.00	0.00	0.00	0.00	0.00
WT S	0.80	0.30	0.00	1.09	0.23
WT +CPT	0.76	0.28	0.11	1.15	0.28

Supplementary Table 4. Yeast strains used in this study.

Strain	Genotype
YBP-275 (I-SceI)	*MATa-inc, bar1Δ, ade2-1, can1-100, leu2::SFA1, trp1-1, ura3-1, lys2::GAL1p-ISCEI, adh4::URA3::GAL1p::leu2Δ3'::ACT1iΔ3'::IsceIsite his3::HYG:HOsite::ACT1-iΔ5'::leu2Δ5'*
WT	*MATa, ade2-1, trp1-1, can1-100, leu2-3,112, his3-11,15, ura3; GAL, psi+, RAD5; URA3::GPD-TK(7x)*
pif1-m2	*MATa, ade2-1, ura3-1, his3-11,15, leu2-3, 112, trp1-1, CAN1, GAL, PSI+, sml1:TRP1, pif1-m2*
