# Supplementary material for: qDSB-Seq is a general method for genome-wide quantification of DNA double-strand breaks using sequencing
Source: Nat Commun. 2019 May 24;10:2313. doi: 10.1038/s41467-019-10332-8 (PMC6534554; doi:10.1038/s41467-019-10332-8)
Supplement: Supplementary file 3 — Description of Additional Supplementary File [file 41467_2019_10332_MOESM3_ESM.pdf]

Description of Additional Supplementary Files

Supplementary Data 1. Restriction enzymes cutting sites for *Saccharomyces cerevisiae*, *Homo Sapiens*, *Mus musculus*, *Drosophila melanogaster*, *Arabidopsis thaliana*, and *Caenorhabditis elegans*.
